# Quackery Rampant

**Published:** 1860-08

**Authors:** 


*Quackery Rampant.*—We have recently seen a case in the hands of Dr. Beard, of this city, which exhibits in the most glaring manner the extraordinary credulity of man. The reader must know that in this city we have a wonderful "negro doctor," to whom the sick of high and low degree flock for relief. If ignorance ever was sublimated and concentrated, it is so in this miserable old African, and yet he is worshipped as an oracle by many who would be angry if we called them fools. The case we particularly allude to is one of double cataract in a highly respectable old planter. To look into his face is to diagnosticate his disease, so strikingly manifest is it. Yet during two long months has he been in the hands of this old negro, who day by day has been *blowing a powder into his eyes* (almost literally "throwing sand into his eyes") thereby complicating the case by irritating the conjunctiva. Yesterday Dr. B. operated on both eyes, and to-day vision is quite distinct. There can be no doubt that in a few days he will be walking our streets without the aid of friends. His first visit ought to be to the old "negro doctor." He ought to behold the object on which he reposed such confidence.

Thus it is, from year's end to year's end, our city is infested with these miserable imposters; though, thanks to Yellow Jack, few remain here during the summer. The winter before last "Dr. Ealing" cured (!!) the corns of our gentry to the tune of thousands, and last winter Dr. Don Muskwhiskers poked his fingers into their eyes and ears—to say nothing of his operations on their pockets. We earnestly hope they will both return in the fall. All the fools are not dead. and their presence is indispensable.—*N. O. Med. News.*

